# Identification of a novel linear B-cell epitope using a monoclonal antibody against the carboxy terminus of the canine distemper virus nucleoprotein and sequence analysis of the identified epitope in different CDV isolates

**DOI:** 10.1186/s12985-017-0858-6

**Published:** 2017-09-29

**Authors:** Li Yi, Zhigang Cao, Mingwei Tong, Yuening Cheng, Yong Yang, Shuang Li, Jianke Wang, Peng Lin, Yaru Sun, Miao Zhang, Shipeng Cheng

**Affiliations:** 1 Institute of Special Wild Economic Animal and Plant Science, Chinese Academy of Agricultural Sciences, Changchun, China; 2 Key Lab of Zoonosis Research, Ministry of Education Institute of Zoonosis, Jilin University, Changchun, China

**Keywords:** Canine distemper virus, Nucleoprotein, Monoclonal antibody, B-cell epitope

## Abstract

**Background:**

The Nucleoprotein (NP) is the most abundant and highly immunogenic protein in canine distemper virus (CDV), playing an important role in CDV viral replication and assembly.

**Results:**

In this study, a specific monoclonal antibody, named C8, was produced against the NP protein C terminal (amino acids 401–523). A linear N protein epitope was identified by subjecting a series of partially overlapping synthesized peptides to enzyme-linked immunosorbent assay (ELISA) analysis.The results indicated that ^444^GDKYPIHFNDER^455^ was the minimal linear epitope that could be recognized by mAb C8. Sequence alignments demonstrated that this linear epitope is less conserved among three CDV genotypes. We next analyzed the level of conservation of the defined epitope in19 Chinese CDV clinical isolates, and it has one site variation in amino acid among these CDV isolations. 2 isolates have the amino acid mutations F451L, while one has P448Ssubstitution.Phylogenetic analysis showed the two isolates with F451Lsubstitution had a closer relationship in a virulent strain ZJ-7, so the epitope may be a significant tag associated with virus virulence.

**Conclusion:**

This collection of mAb along with defined linear epitope may provide useful reagents for investigations of NP protein function and the development of CDV specific diagnostics.

## Background

Canine distemper virus (CDV) is an enveloped negative-strand RNA virus classified into the genus *Morbillivirus* within the family *Paramyxoviridae* [1]. CDV infection results in systemic disease with involvement of the central nervous system and the respiratory and gastrointestinal tracts [2]. With the rapidly development of fur-animal industry and the expansion number of dogs and fur animals, the economic loss is significant due to CDV infection, especially in mink and fox farms in China [3]. Canine distemper (CD) disease has raised global attention with its extensively spreading.

Nucleoprotein (NP) is the most abundant and highly immunogenic protein in *Morbillivirus*, and is an internal protein which packs the viral RNA genome and forms a helical nucleocapsid. It can be used as the basis of most diagnostic assays for CDV presently [4]. The interest in diagnostic applications of CDV NP has focused attention on identifying more antigenic epitopes, especially B cell epitopes [5]. However, the data on B cell epitopes of nucleoprotein are still limited, especially in the C-terminal region (aa 401–523).The objective of this study was to identify B cell epitopes in CDVNP C-terminal region using monoclonal antibodies. Additionally, the high degree of variation in NP C-terminal region can be used to classify CDV into different genotypes [6]. The epitope identified in this area may be diverse and may be used as a tag to distinguish different CDV isolates. Therefore, we decided to perform the specific mAbs and identify their epitopes in CDVNP C-terminal region precisely.

In this study, we generated one CDV NP-reactive mAb and mapped its linear B-cell epitope. We also used bioinformatics tools to locate linear B-cell epitope within the CDV NP C-terminal region (aa 401–523) to a predicted 3-dimensional (3D) model. These results will facilitate the development of epitope-based preventive and diagnostic strategies for CDV infection and provide resources for better understanding the antigenic structure and function of the CDV NP protein.

## Methods

### Ethics statement

The protocol of this study was carried out in accordance with guidelines of animal welfare of World Organization for Animal Health. All experimental protocols were approved by the Review Board Institute of Special Animal and Plant Sciences, Chinese Academy of Agricultural Sciences (CAAS).

### Cell lines and viruses

Vero and the SP2/0 myeloma cell line were cultured in Dulbecco’s Modified Eagle’s Medium (DMEM, Invitrogen) in a humidified incubator with 5% CO_2_ at 37 °C. media for Vero cells and SP2/0 myeloma cells were supplemented with 10 and 20% heated-inactivated fetal bovine serum (GIBCO, Invitrogen) along with antibiotics (0.1 mg/ml of streptomycin and 100 IU/ml of penicillin), respectively. The CDV-PS [7] strain (JN896331.1) was lab-adapted and was propagated in Vero cells using standard techniques.

### Cloning of the CDV NP C-terminal region (aa 401–523) and prokaryotic expression of recombinant protein

Vero cells were infected with the lab-adapted CDV-PS strain. At 40–50% cytopathic effect, the cells were lysed and total RNA was extracted by using an RNeasy RNA extraction kit (Qiagen) according to the manufacturer’s instructions. Following RNA extraction, cDNA was synthesized by reverse transcription using a First Strand cDNA synthesis kit and random primers (Roche Inc., Germany). This cDNA was used to amplify the C-terminal region (aa 401–523) of the N gene by using the forward and reverse primers CDV N-KpnI-aa401 (5′-CC*GGATCC*GGTGCAGTTGCAC-3′) and CDV N-SacI-aa523 (5′-GC*GAGCTC*ACCTTCGGTAA-3′). The PCR product was cloned into the pET32a(+) expression vector (Novagen, USA) by double digestion with *Kpn*I and *Sac*I. Plasmid construction was verified by restriction enzyme digestion, PCR, and DNA sequencing. The recombinant plasmid, named pET-N-c, was transformed to *E. coli* to produce recombinant protein fused with a six-histidine tag. Recombinant protein was induced by adding isopropyl-D-thiogalactopyranoside (IPTG). For expression, IPTG was added to a final concentration of 0.75 mM and cells were incubated for 6 h at 37 °C. Production of recombinant protein was evaluated by sodium dodecylsulfate-polyacrylamide gel electrophoresis (SDS-PAGE). The recombinant protein was purified by affinity chromatography using a His-Tag resin according to manufacturer’s instruction (Roche Inc., Germany). Production of the purified recombinant protein was confirmed by Western Blot using CDV-positive dog serum as follows. Recombinant protein was boiled 5–10 min, separated by SDS-PAGE, and transferred to nitrocellulose membranes. The membranes were blocked with 5% skim milk overnight at 4 °C to reduce non-specific binding. The membrane was incubated with CDV-positive dog serum as the primary antibody at a 1:100 dilution. After incubation, each was washed five times with PBST, and then it was treated with HRP-conjugated rabbit anti-dog secondary antibodies (Bioss Inc., China). The color was developed using 3,3′-diaminobenzidine tetrahydrochloride (DAB) and stopped by rinsing in deionized water followed by drying the membrane.

### Preparation and identification of mAb against CDV recombinant N protein (401-523aa)

The truncated N protein (401-523aa) was used as an antigen to immunize mice to generate CDV N-specific mAbs according to standard procedures [8]. Six-week-old female BALB/c mice were immunized subcutaneously with 100 μg recombinant protein as above. After 14 days, mice received a subcutaneous booster immunization consisting of 200 μg purified protein. The mice received a final booster immunization 3 days prior to harvesting splenocytes for hybridoma generation. Splenocytes were fused with SP2/0 myeloma cells at a ratio of 4:1 using polyethylene glycol (PEG4000, Sigma-Aldrich). The cells were seeded into 96-well plates in HAT medium (DMEM containing 20% FBS, 0.1 mg/ml streptomycin, 100 IU/ml penicillin, 100 mM hypoxanthine, 16 mM thymidine, and 400 mM aminopterin) for 6 days at which time the media was replaced with HT medium (HAT medium is lacking aminopterin). After HAT/HT selection, culture supernatants of surviving clones were screened for reactivity and specificity by indirect ELISA, WB and IFA.

For WB, the MAb was analyzed by western blotting with Vero cells that had been infected with CDV-PS. Each sample was separated by 12% SDS-PAGE, and transferred onto nitrocellulose membranes with a Transblot apparatus (Bio-Rad, USA). The membrane was incubated individually with the appropriate dilution of the mAbs, and then incubated with a 1:4000 HRP-conjugated rabbit anti-mouse antibody. Immunoreactive bands were visualized using DAB Western Blotting Detection Reagents (CWBIO, China).

For IFA, the 96 well microtiter plate, where Vero cells were cultured, was added with CDV (CDV-PS strain). About 24-48 h later, Vero cells were fixed with 90% (*V*/V) ethylalcohol in pre-cooled distilled water at 4 °C for 30 min. The cells were washed with phosphate buffered saline (PBS, pH 7.4) with 0.1% Tween-20 (Sigma, USA) and were added in hybridoma supernatants for incubating at 37 °C for 1 h. After the plates were washed again, a FITC-conjugated goat anti-mouse IgG (Sigma, USA) was added as secondary antibody. Finally, the plate was viewed by a fluorescence microscope after washing three times.

The mAb subtypes was determined using the Mouse Monoclonal-Ab-ID kit (Invitrogen, CA, USA) as the manufacturer’s instructions.

### Identification of the linear epitope by Pepscan analysis

Twenty-three overlapping synthetic peptides (15aa) corresponding to the coding sequence of recombinant N protein (aa 401–523) were synthesized (Sangon, China) to map the mab binding epitope. The synthetically peptides were 15 amino acid (aa) residues long and each peptide overlapped the previous peptide sequence by 10 aa residues. Peptides plated as coating antigen to assess the mAb binding by Pepscan analysis. Briefly, the ELISA plates were coated with these peptides at 100 ng well^−1^ at 4 °C overnight and then incubated with the culture supernatant of hybridoma cells at 37 °C for 1 h. HRP-conjugated goat anti-mouse IgG was used as the secondary antibodies at a 1:4000 dilution at 37 °C for 1 h, followed by color development with substrate solution containing TMB. Based on the result of this initial screen used to identify a linear region of N protein (401-523aa) recognized by the mAb, we designed and synthesized a series of progressively truncated polypeptides to perform a more refined mapping of the minimal linear peptide epitope recognized by the mAb using the ELISA approach described above.

### Location analysis of identified epitope in the CDV N protein (401-523aa)

In order to locate and explain the general spatial relationship of epitope on CDV N protein (401-523aa) according to the homology modeling from PDB, a 3D model of the protein was generated by using I-TASSER online sever [9, 10]. And the PyMol software was used based on the results of I-TASSER online sever to analyze the epitope locations onto N protein 3D model (401-523aa).

### Homology analysis of defined epitope

To investigate the homology of the epitope to equivalent CDV sequences, alignments of sequences from homologous regions of 17 CDV strains (including CDV Asia-1, Vaccine and America-2 genotype strains) were completed by using the DNASTAR Lasergene program (Windows version; DNASTAR Inc., Madison, WI).

### Phylogenetic analysis of the N gene C-terminal region (aa 401–523) from 19 chinese clinical Canine distemper virus strains

Alignment analysis was also performed between the 19 Chinese CDV clinical isolates, the factors of isolation time and geographical location of Chinese clinical strains were considered (Table 1).

**Table 1:** Characteristics of canine distemper virus in domestic dogs in this study

Strain	Region	Host	Origin	Year	Accession no.	Clinical symptoms
CDV-WH-13	Wuhan	Dog	Nasal swab	2015	KU558767	Coughing, eye discharge
CDV-WH-30	Wuhan	Dog	Nasal swab	2015	KU558768	Eye discharge
CDV-WH-31	Wuhan	Dog	Nasal swab	2015	KU558769	Nasal discharge, vomiting
CDV-WH-33	Wuhan	Dog	Nasal swab	2015	KU558770	Nervous signs
CDV-WH-34	Wuhan	Dog	urine	2015	KU558771	Nasal discharge
CDV-WH-43	Wuhan	Dog	urine	2015	KU558772	Vomiting, diarrhea
CDV-WH-46	Wuhan	Dog	fecal	2015	KU558773	Eye discharge
CDV-WH-56	Wuhan	Dog	fecal	2015	KU558774	Diarrhea
CDV-WH-60	Wuhan	Dog	Nasal swab	2015	KU558775	Nervous signs
CDV-WH-64	Wuhan	Dog	blood	2015	KU558776	Nasal discharge
CDV-WH-72	Wuhan	Dog	Nasal swab	2015	KU558777	Diarrhea
CDV-SH-3	Shanghai	Dog	blood	2016	KU558778	Nasal discharge
CDV-SH-7	Shanghai	Dog	Nasal swab	2015	KU558779	Diarrhea
CDV-CC-4	Changchun	Dog	Nasal swab	2016	KU558780	Nasal discharge
CDV-CC-11	Changchun	Dog	Nasal swab	2015	KU558781	Nervous signs
CDV-CC16	Changchun	Dog	Nasal swab	2015	KU558782	Diarrhea
CDV-CC-27	Changchun	Dog	Nasal swab	2015	KU558783	Nasal discharge
CDV-DL-3	Dalian	Dog	Nasal swab	2016	KU558784	Nasal discharge
CDV-DL-5	Dalian	Dog	Nasal swab	2015	KU558785	Vomiting, diarrhea

RNA was extracted from Vero cells infected with wildtype CDV Chinese strains. The extraction was done as indicated above. The first-strand cDNAs were synthesized using random primers. For each virus, the coding sequence of the C-terminal region (aa 401–523) was amplified by RT-PCR using forward and reverse primers as indicated above. To assess the status of relationship among representative clinical CDV viruses, we built a CDV phylogenetic tree of nucleocapsid protein genes which contains the identified CDV N linear epitope. The C-terminal part of the nucleocapsid protein genes from the 19 sampled dogs were assembled and edited over a total length of 372 bp using the SeqMan program (Lasergene7.1, DNASTAR Inc., Madison, WI).Subsequently, these sequence data were submitted to GenBank, and the accession numbers are shown in Table 1. For comparative analysis, the nucleotide sequences of 13 CDV strains available from GenBank, including the 19 sequences characterized here, were aligned using the Clustal W program of MEGA 5 software, and a phylogenic tree was constructed using the neighbor-joining (NJ) method. The robustness of the phylogenetic analysis was assessed by a bootstrap analysis with 1000 replicates.

## Results

### Prokaryotic expression and purification of recombinant N protein (C-terminal 401-523aa)

**Fig. 1:**
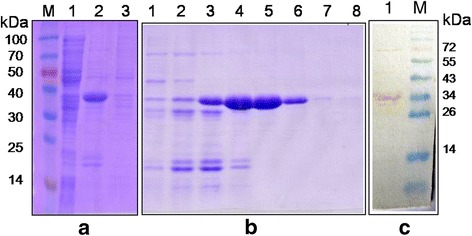
Expression and purification of recombinant CDV N protein (C-terminal 401-523aa). **a** SDS-PAGE analysis of the recombinant N protein (C-terminal 401-523aa).Lane M, molecular weight marker; Lane 1, proteins expressed in *E. coli* without IPTG induction. Lane 2, supernatant of *E. coli* cell lysates expressed with IPTG induction; Lane 3, inclusion bodies of *E. coli* cell expressed with IPTG. **b** SDS-PAGE analysis of the purified N protein (C-terminal 401-523aa). lane 1–8, purified recombinant protein with different iminazole concentrations: 20 mM, 40 mM, 60 mM, 80 mM, 120 mM, 160 mM, 180 mM, and 200 mM. **c** Western blot analysis of purified protein.Lane M, molecular weight marker; Lane 1, the binding of purified recombinant protein with CDV-positive dog sera

The recombinant N protein (C-terminal 401-523aa) fused with a six-histidine tag was successfully expressed in *Escherichia coli* BL21 (DE3). The recombinant protein was predominantly found within the soluble fraction of the induced *E. coli* after ultra-sonication (Fig. 1a) and was subsequently purified by amylose resin affinity chromatography (Fig. 1b). The recombinant protein was recognized by CDV-positive dog serum in Western blot (Fig. 1c). The expected size of the expressed recombinant protein was about 35 kD for 123 amino acids in NP protein (about 15kD) and expression vector tag (about 20 kD). And the approximate size of the expressed protein was as the same as the expected size.

### Preparation and identification of mAb against CDV N protein

We immunized BALB/c mice with purified the recombinant protein to generate hybridoma lines secreting antibodies against the CDV N protein. Hybridoma cell supernatants were screened by indirect enzyme-linked immunosorbent assay (ELISA) and selected hybridoma lines were subcloned by limiting dilution (data not shown). One hybridoma line stably producing antibodies against CDV N protein were obtained and named C8.

**Fig. 2:**
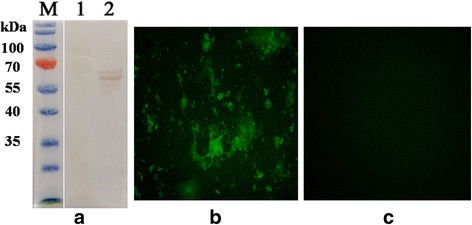
Characterization of CDV N-reactive mAb. **a** The immunoreactivity of native CDV N protein to MAb was analyzed with Western blotting. Lane M, PageRuler™ Prestained Protein Ladder (Invitrogen); Lane 1, Cell lysates from uninfected Vero cells; Lane 2, Lysate of Canine distemper virus-infected Vero cells. **b** Vero cells were infected with CDV3 and used to evaluate mAb reactivity by IFA. **c** Uninfected Vero cells represent the negative control

Western blot analysis revealed that mAb produced by hybridoma line reacted with the native N protein derived from CDV3-infected Vero cells, but did not recognize uninfected Vero cell lysates (Fig. 2a). In addition, the mAb was able to react with Vero cells infected with CDV3 (Fig. 2b) by indirect immune-fluorescence assay (IFA), but did not bind appreciably to uninfected Vero cells (Fig. 2c).

### Identification of B-cell epitope recognized by CDV carboxy terminus of N protein mAb

**Fig. 3:**
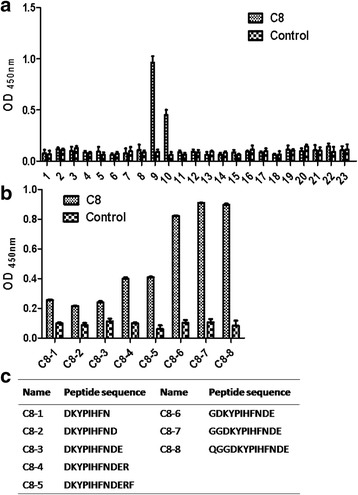
Identification of minimal linear epitope recognized by mAb C8. **a** C8 was screened by indirect ELISA against a panel of 23 overlapping peptides derived from the CDV nucleoprotein protein (aa 401–523). The SP2/0 cell culture supernatant was used as the negative antibody control. The error bars display the standard deviation of three experimental repeats. **b** Mab C8 was screened against a series of truncated peptides, with amino acid residues progressively deleted from the amino or carboxyl terminus, to determine the minimal amino acid sequences required for MAb binding. The SP2/0 cell culture supernatant was used as the negative antibody control. **c** The name and sequence of the peptides used are included in the table

We next sought to define the linear epitopes within the CDV N protein (C-terminal 401-523aa) recognized by the mAb. Peptide scanning technology was used to prepare a group of 23 overlapping synthetic peptides (15 amino acids [aa]) corresponding to the truncated N protein (C-terminal 401-523aa). Each 15 aa synthetic peptide overlapped the previous peptide sequence by 10 aa residues. The synthetic peptides were respectively used as coating antigen in an indirect ELISA to identify the epitope recognized by the C8 mAb. The C8 mAb recognized both synthetic peptides ^440^ENQGGDKYPIHFNDE^454^ and ^445^DKYPIHFNDERFPGY^459^ (Fig. 3a), suggesting that the core linear epitope might be represented by the sequence ^445^DKYPIHFNDE^454^which was the overlapping N protein sequence present in both peptides. To define the minimal linear peptide epitope required for mAb recognition, we designed and synthesized a series of progressively truncated polypeptides starting with the initial peptides recognized by the mAb as described above. Amino acids were progressively deleted from the C- or N-terminus of the two peptide sequences (^445^DKYPIHFNDERF^456^and ^442^QGGDKYPIHFNDE^454^) respectively. The minimal peptide epitope recognized by mAb C8 was ^444^GDKYPIHFNDER^455^ (Fig. 3b).

### Location analysis of B cell identified epitope in the CDV N protein (C-terminal 401-523aa)

**Fig. 4:**
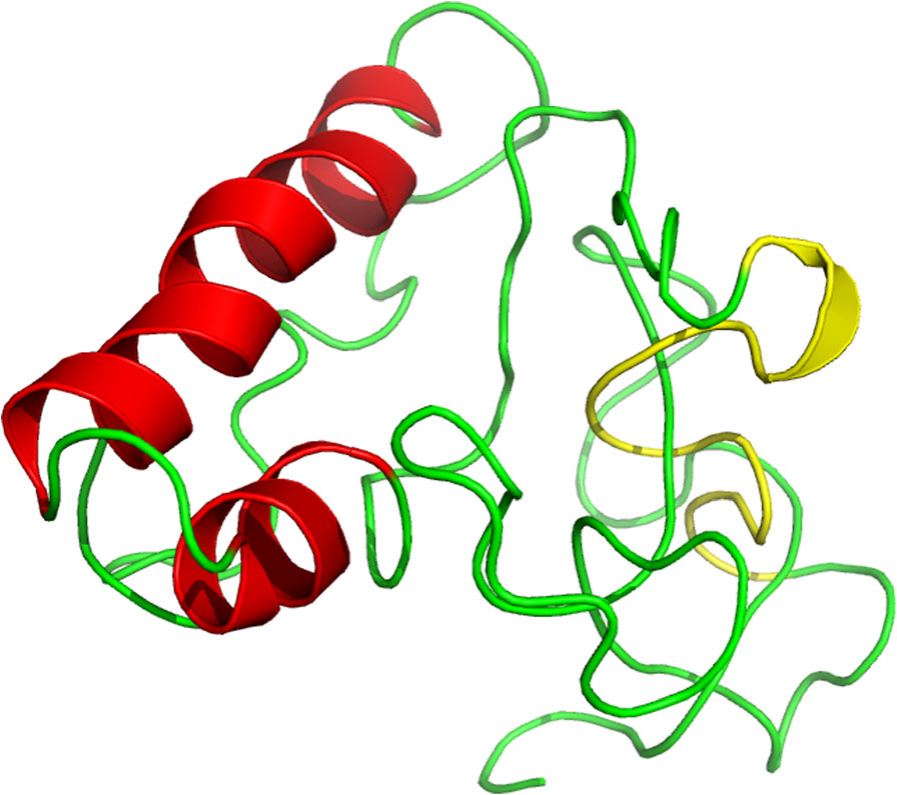
Structural location analysis of epitope on the CDV N protein (C-terminal 401-523aa). Location analysis of the identified B cell epitope using PyMol software. 3D homology modeling was used by I-TASSER online sever. The epitope 444GDKYPIHFNDER 455 was labled and shown by *yellow*, and the helix locations were shown by *red*

Because the 3D structure of CDV N protein (C-terminal 401-523aa) was not available in Protein Data Bank (PDB), several 3D homology modeling about it was obtained from I-TASSER online sever. Then we had chosen one of them according to high confidence that corresponding with the information of secondary structure of N protein (C-terminal 401-523aa).The locations of identified epitope was showed in the context of the deduced 3D protein model (Fig. 4).

### Alignment and conversation analysis of the defined epitope

We next analyzed the level of conservation of the defined epitope among different CDV genotypes.We aligned sequences corresponding to the region encompassing the mAb C8 epitope (aa 444–455) from the CDV isolates for which sequence information was available from the NCBI protein database (http://www.ncbi.nlm.nih.gov/protein).Alignment of the amino acid sequences revealed that the peptide epitope recognized by mAb 1 N8 is not conserved among CDV isolates, which has several substitutions in genotype Asia-1, Vaccine and America-2 strains (Table 2).The CDV genotypeAmerica-2 and Vaccine strains (Onderstepoort and Vaccine Hungary) displayed a single N➔S substitution at amino acid position 452. The other CDV Vaccine strains (Lederle and CDV3) displayed K➔R and N➔S substitutions at amino acid position 446 and 452. K446R and N452S substitutions were always observed in the genotype Asia-1 strains, while the Y447S, P448S and F451L/I/V substitutions were also observed.

**Table 2:**
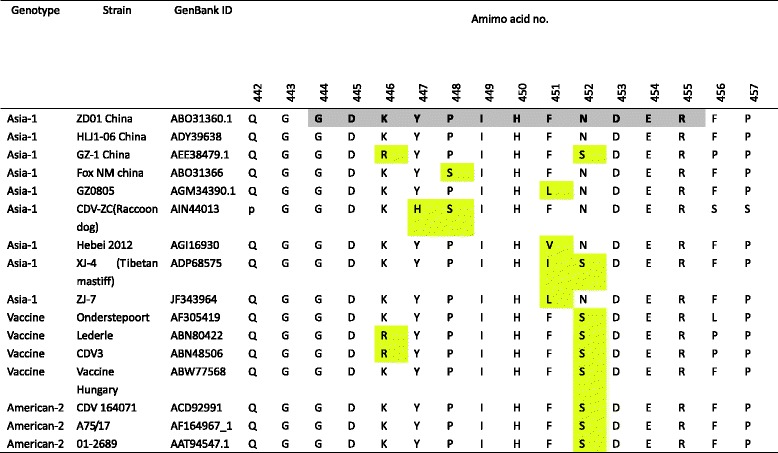
Conservation analysis of the epitope C8 among different CDV strain

The yellow highlight indicated the amino acid identity and differences among different CDV strains

### The defined epitope alignment between Chinese CDV clinical isolates

**Fig. 5:**
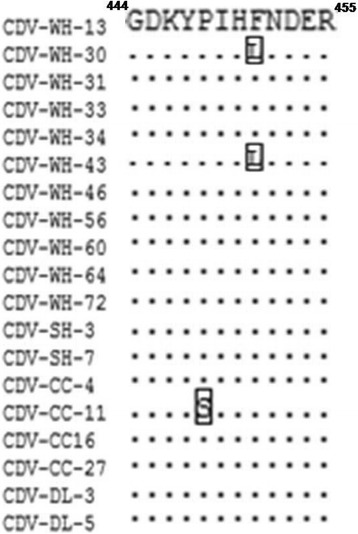
Amino acid alignments of the identified epitope region from CDV Chinese isolates in this study

To assess the degree of conservation of the linear epitope in Chinese clinical isolates, we analyzed the CDV N protein (C-terminal 401-523aa) sequences from Chinese isolates in domestic dogs during 2015–2016.Analysis of these sequences from 19CDVclinical isolates indicated that the C8 epitope,^444^GDKYPIHFNDER^455^ is highly conserved among CDV Chinese strains.Limited amino acid mutations were present in three CDV strains, whereas an F➔L substitution at position 451 was noted in CDV-WH-30, CDV-WH-43 and an P➔S substitution at position 448 was noted in CDV-CC-11 (Fig. 5).

### Phylogenetic analysis of CDV C-terminal part of the nucleocapsid protein gene

**Fig. 6:**
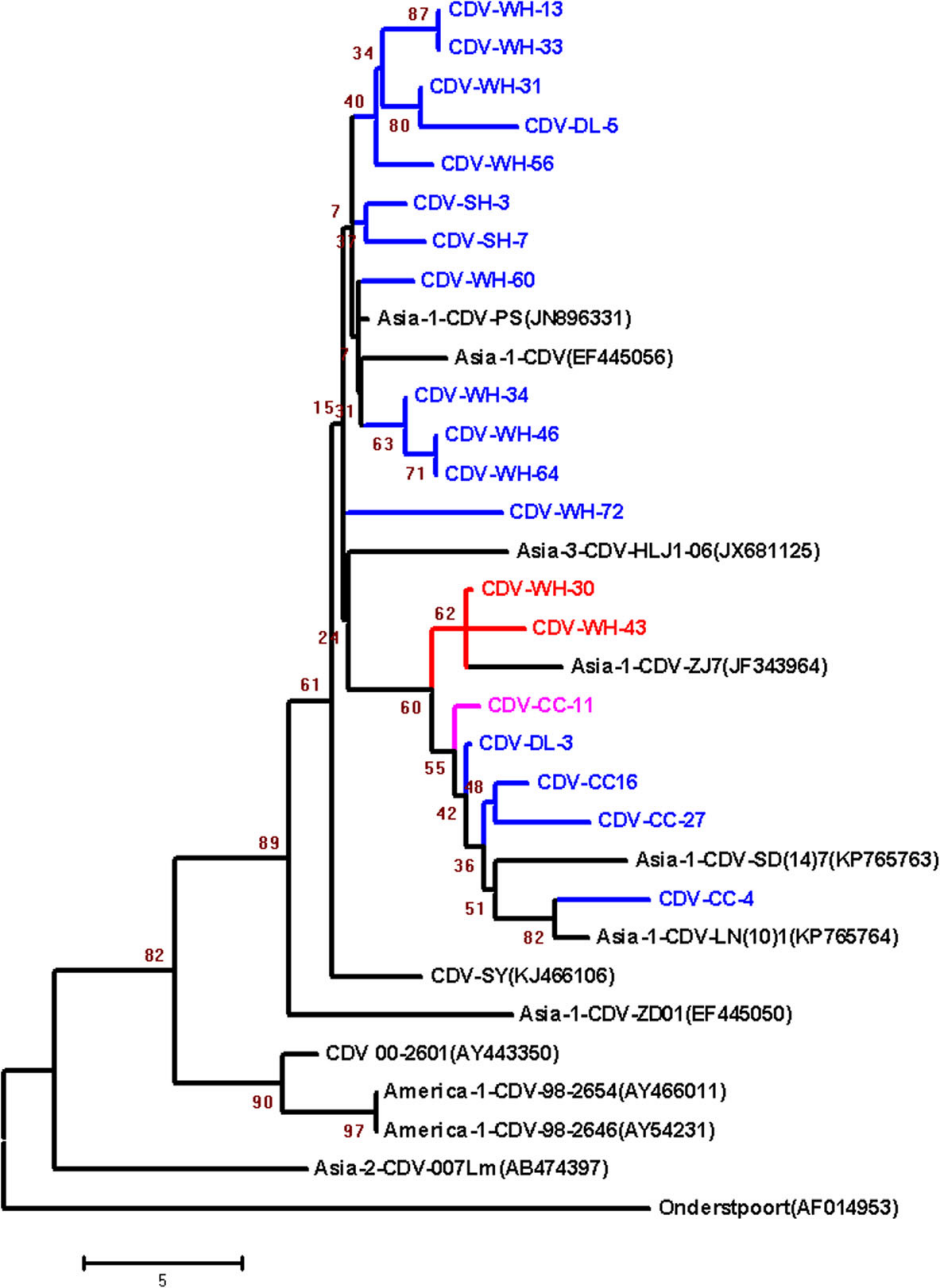
Neighbor-joining phylogenetic tree analysis. The nucleotide sequences of carboxy terminus of nucleocapsid genes come from representative international (GenBank accession numbers detailed in Figure) and Chinese CDV isolates. Bootstrap support was calculated using 1000 data replicates. Horizontal branch lengths are proportional to genetic distances. Branches are colored according to the identified epitope state ^444^GDKYPIHFNDER^455^, the epitope has no mutation (*blue clade*), one substitution with P448S (*pink clade*) and F451L (*red clade*)

Phylogenetic analysis showed that the 19 CDV strains were separated into two groups. The strains WH-13, WH-33, WH-31, WH-56, WH-60, DL-5, SH-3 and SH-7 displayed high sequence identity to each other and were grouped in one clade along with some CDV strains from China such as CDV-PS (JN896331). The other strains WH-30, WH-43, CC-11, DL-3, CC-16, CC-27 and CC-4, formed another single branch sharinghigh nucleotide sequence identity with Chinese strain ZJ-7 (JF343964) which is a high virulent strain. The strains WH-30 and WH-43, including one substitution with F451L in the identified epitope, had a close evolutionary relationship tostrain ZJ-7 (JF343964).The strain CC-11, with one substitutionP448S in the epitope, shared a higher variation and formed in a single branchindividually, which seems like the ancestor of strains SD(14)7 and LN(10)1 (Fig. 6).

## Discussion

The *Morbillivirus* nucleoprotein is an important structural protein playing the role of virus assembly and has some regulatory functions in viral transcription and replication [11].

Recent structural studies on NP within the genus Morbillivirus have revealed that it consists of three regions: the highly conserved central region, the poorly conserved N- and C-terminal domain. The N-terminal region, with conserved sequences required for self-assembly and RNA binding; and a C-terminal region, which is intrinsicallydisordered and protrudes from the viral nucleocapsid surface [12, 13]. Regions of binding with P protein in CDV have recently been mapped within the NP C-terminal region, in agreement with the accessibility of C-terminal region on the surface of the nucleocapsid [14]. Localization of B cell epitopes may provide additional information on the structure of CDV NP C-terminal region. A detailed analysis of the antibody epitope within this area would further our understanding of CDV immunity and the development of epitope-based marker vaccines and diagnostic tools.

Moreover, this is the first report of an epitope (^444^GDKYPIHFNDER^455^) in the CDV NP C-terminal region, which can be potentially valuable for further CDV research.

In this study, we used prokaryotic recombinant protein HIS-NP(C-terminal) for the immunization of BALB/c mice and screening hybridoma supernatants for NP-reactive antibody production to exclude potential false positive results. One CDV NP-reactive mAb that recognized NP protein produced by CDV-PS strain was generated.

We screened a panel of CDV N protein (C-terminal 401-523aa) overlapping synthetic peptidesto map mAb reactivity to initial B cell linear epitope. And the progressively truncated peptides were synthesized to define the minimal B cell linear epitope recognized by the mAb. In the initial screen, mAb C8 reacted with twosynthetic peptides which overlapped by 10 amino acids. The binding of C8 to peptide ^440^ENQGGDKYPIHFNDE^454^ resulted in a twice higher OD450 value when compared to C8 binding to ^445^DKYPIHFNDERFPGY^459^, suggesting that the overlapping sequence was sufficient for C8 binding, but additional residues present in ^445^DKYPIHFNDERFPGY^459^ enhanced binding. A peptide lacking the C-terminal ^444^G residue of this core peptide sequence displayed a strong reduction in C8 binding. When residues at the C-terminus were progressively added, no more increment in C8 binding was noted until the residue ^455^R was added. Therefore, we demonstrated that ^444^G and ^455^R were critical components of the linear epitope.

Based on previous research [15], the NP C-terminal region appears to be a tail extending from the surface of the globularbody. We also mapped the location of identified epitope on 3D homology model of NP protein C-terminal region in this study. There is no C-terminal ends of the NP protein crystal structure available now, so the I-TASSER online sever was used to predict the 3D structure based on different templates of high confidence by sending the primary sequence of NP protein (401-523aa). In this accord, the B-cell epitope recognized by the mAb is predicted to be located at the surface of the protein within a kind of helix structure.The analysis of the protein structure could potentially facilitate the function study of the NP C-terminal protein.

Alignment of NP amino acid sequences of different CDV genotypes and CDV clinical isolates in China showed that the defined antibody epitope isless conserved among CDV genotypes, which has several substitutions in genotype Asia-1, Vaccine and America-2 strains. The N452S substitution is a solid change in CDV genotype America-2 and Vaccine (America-1).There are another substitutions in this defined epitope, which demonstrates less conserved between different CDV strains. To assess the degree of conservation of the linear epitope, we analyzed these sequences from Chinese isolates in domestic dogs during 2015–2016. Three out 19 (15.7%) amino acid mutations were present in Chinese CDV strains, which are F451L and P448S. Interestingly, the F451L substitution also can be observed in the strain ZJ7 isolate [16] (JF343964) which is a high virulent strain. Moreover, the two isolates with F451L substitutionformed a single branch sharing high identity with Chinese strain ZJ-7 in the phylogenetic tree based on C-terminal part of the nucleocapsid protein genes, so the F451L substitution in CDV NP protein maybe a tag which can identified as a high virulent strain. On the other hand, the CDV NM strain, CDV-ZC and XJ-4 [17] stain show some substitutions in this identified epitope, while the hosts of viral infection are fox, raccoon dog and Tibetan mastiff, respectively. There may be a relationship between host range and this epitope.

## Conclusions

The identification of less conserved epitope within the NP protein (401-523aa) of CDV could provide important insights into the structure and function of the NP protein. Moreover, the defined epitope can be used to design suitable serological methods for epidemiological surveillance, and may suggest potential targets for pharmacological inhibitors. The established MAb and defined epitope could be used to further study the structure and function of NP protein during CDV infection and provide a foundation for the development of novel epitope-based vaccines.
